# Viral Gastroenteritis Associated with Genogroup II Norovirus among U.S. Military Personnel in Turkey, 2009

**DOI:** 10.1371/journal.pone.0035791

**Published:** 2012-05-11

**Authors:** Salwa F. Ahmed, John D. Klena, Manal Mostafa, Jessica Dogantemur, Tracy Middleton, James Hanson, Peter J. Sebeny

**Affiliations:** 1 United States Naval Medical Research Unit No.3, Cairo, Egypt; 2 Incirlik Air Base, Incirlik, Turkey; Duke-NUS Gradute Medical School, Singapore

## Abstract

The present study demonstrates that multiple NoV genotypes belonging to genogroup II contributed to an acute gastroenteritis outbreak at a US military facility in Turkey that was associated with significant negative operational impact. Norovirus (NoV) is an important pathogen associated with acute gastroenteritis among military populations. We describe the genotypes of NoV outbreak occurred at a United States military facility in Turkey. Stool samples were collected from 37 out of 97 patients presenting to the clinic on base with acute gastroenteritis and evaluated for bacterial and viral pathogens. NoV genogroup II (GII) was identified by RT-PCR in 43% (16/37) stool samples. Phylogenetic analysis of a 260 base pair fragment of the NoV capsid gene from ten stool samples indicated the circulation of multiple and rare genotypes of GII NoV during the outbreak. We detected four GII.8 isolates, three GII.15, two GII.9 and a sole GII.10 NoV. Viral sequences could be grouped into four clusters, three of which have not been previously reported in Turkey. The fact that current NoV outbreak was caused by rare genotypes highlights the importance of norovirus strain typing. While NoV genogroup II is recognized as causative agent of outbreak, circulation of current genotypes has been rarely observed in large number of outbreaks.

## Introduction

Human noroviruses (NoV) are the most common cause of outbreaks of acute gastroenteritis, worldwide [1]. Annually, NoV have been estimated to be implicated in 21 million cases of acute gastroenteritis in the United States [2], and is the most common cause of diarrhea in adults [3]. One in fourteen people will have a norovirus infection each year, and most people will have suffered several NoV infections within their lifetime [2]. Worldwide, it has been estimated that over 218,000 of pediatric deaths that are attributed to gastrointestinal disease each year from a total of 1.6 million can be directly attributed to NoV illness [3]. Most children will have at least one NoV infection within the first 5 years of life [4]. Sporadic NoV infection has been reported as second to rotavirus as an enteric pathogen associated with gastroenteritis among children [5].

Norovirus is recognized as a major cause of gastroenteritis outbreaks in military populations [6], and other semi-closed or closed institutions such as hospitals, elder care homes, cruise ships, and nurseries [7]. Norovirus strains were a major cause of outbreaks and sporadic cases of gastroenteritis among US ground troops during the Persian Gulf War in 1991, where NoV infection was in one report the most common cause of disability [6] as well as coalition forces in Gulf War II and the Afghanistan campaign beginning in 2002 [8], [9], [10], [11]. As many as 73% of enteric viral gastroenteritis cases among British troops deployed from 2002–2007 were associated with NoV [9]. In 2002, a NoV outbreak among British forces deployed to Afghanistan forced medical evacuations to European hospitals because of severe dehydration and other complications, resulting in closure of the field hospital, leaving the remaining military contingent without full medical services until the outbreak could be characterized [8]. Studies from an array of military units continue to demonstrate that NoV outbreaks have the capacity to impede force readiness and have even caused the stand down of the entire air wing of a battle group [12].

Norovirus belong to the family *Caliciviridae* and have a single-stranded, positive-sense RNA genome that is genetically highly variable, with five known genogroups [1], three (GI, GII, GIV) of which are associated with human disease [1]. The GII NoV are most commonly associated with human disease, with 19 known genotypes [13]; strains of genotype GII.4 in particular are recognized as a dominant circulating strain. Between 1995 and 2006, genotype GII.4 caused at least four pandemic seasons during which novel GII.4 variants rapidly emerged and displaced previously circulating strains [14]. Because of the dramatic increase in GII.4 NoV outbreaks in the past several years, much of the attention with respect to NoV control has focused on strains of this genotype. However, other NoV strains are always circulating, causing local and regional outbreaks [15].

In May 2009, a significant increase in acute gastroenteritis (AGE) cases was noted in the American health clinic at Incirlik Air Base (IAB) in Adana, Turkey. This increased rate of AGE led to discussions with local Turkish military public health authorities, which confirmed that the Turkish military community and the residents of Adana were also experiencing an anecdotal increase in AGE illnesses [16]. An epidemiologic investigation was launched to attempt to identify the cause and possible source of this AGE outbreak at IAB.

## Materials and Methods

### Clinical and Epidemiologic Methods

The current investigation describes a gastrointestinal outbreak which occurred on a US military base in Turkey. The main objective was to identify the etiological agent(s) responsible for the outbreak as a public health response to formulate containment strategy and provide data that would support an appropriate intervention control strategy. The project was conducted as an activity not involving human research, therefore IRB review was not required. Participants voluntarily agreed to provide stool samples and complete the questionnaire information.

IAB is located in the village of Incirlik, about six miles from the city of Adana in southeastern Turkey. It is a Turkish military complex, composed of both Turkish and American personnel. The American component has approximately 1250 active duty personnel and 1300 civilians and dependents. The vast majority of U.S. military and their dependents live and work on the base, but travel within and outside Turkey is common for base personnel and their families.

Due to a rapid increased report of AGE in the local community and anecdotal cases reported among US personnel at IAB in May, 2009, a clinic case-based investigation was performed. The goal of this investigation was to identify whether an outbreak was underway and if so, to determine the scope of the outbreak, the etiologic agent and its source, in order to mitigate risk and minimize impact as necessary. Patients presenting with diarrhea were requested to fill in a brief self-completed survey starting on 18 May, 2009. The survey collected demographic information, clinical signs and symptoms, and risk factors. Patients were asked to voluntarily submit a diarrheal stool sample for laboratory analysis. The case-based investigation ended on 18 June, 2009 as gastroenteritis cases dropped to a frequency typical for summer.

To identify the frequency of patients presenting with AGE just prior to the beginning of the presumed outbreak and continuing over time (from May 17–June 27, 2009), data from the clinic’s electronic medical record was utilized by public health officials. This was done through identification of cases with a disease non-battle injury (DNI) code of ‘gastrointestinal, infectious’ which encompasses AGE, to characterize the likely onset and duration of the outbreak.

### Microbiological Analysis

Stool samples were collected in clean stool hats and transferred into a sample cup and stored at 4°C until initial testing. An aliquot of each sample was tested locally using an ELISA for NoV (IDEIA Oxoid (Ely) Ltd, Denmark House, Angel Drove, UK). An additional aliquot of each specimen was also tested by EIA for *Cryptosporidium parvum*, *Entamoeba histolytica*, and *Giardia lamblia (*Techlab Inc., Blacksburg, VA, USA). The remaining sample was stored in Cary-Blair transport medium until transferred to the enteric disease research laboratory at US Naval Medical Research Unit No.3, Cairo, Egypt. In Cairo, standard bacterial culture investigation was performed to detect *Campylobacter* spp., *Salmonella* spp., *Shigella* spp., and *Vibrio* spp. Bacterial isolates were identified and confirmed using standard biochemical methods, and antibiotic sensitivity was determined via disk-diffusion (Becton Dickinson, Cockeysville, MD, USA), and/or E-tests (AB- Biodisk, Sölna, Sweden) as guided by the Clinical Laboratory Standards Institute (CLSI) [17]. EIA was also performed to test for astrovirus, rotavirus, adenovirus, and repeated for NoV (IDEIA, Oxoid, UK) according to the manufacturer’s instructions. DNA was extracted from *Escherichia coli*-like colonies grown on MacConkey-lactose medium and subsequently tested for the enterotoxin genes *eltB* and *estA* [18].

### RNA Extraction, RT-PCR and cDNA Sequencing

Viral RNA was extracted from 140 µl of a 10% fecal suspension in vertrel/water solution using a QIAamp Viral RNA Mini Kit (QIAGEN, Valencia, CA, USA) according to the manufacturer’s protocol. Identification of NoV was performed by RT-PCR as previously described [19]. Genogroup II NoV was identified using primers targeting GII-specific regions in the 5′ capsid gene as described [20]. PCR amplicons from NoV-positive specimens were sequenced using the ABI Prism Big Dye Terminator cycle sequencing kit and an ABI Prism 3100 DNA sequence (Applied Biosystems, Foster City, CA, USA). Sequences were aligned against NoV capsid sequences obtained from Genbank using BioEdit software, version 5.1. Phylogenetic analysis was carried out using MEGA 5.0 [21] and a dendrogram was constructed using the neighbor-joining method with genetic distance modeled using the Kimura 2-parameter model [22]. Statistical confidence for the phylogenetic branches was assessed by bootstrap analysis (2,000 replicates) [23]. The tree is drawn to scale, with branch lengths in the same units as those of the evolutionary distances used to infer the phylogenetic tree [24].

## Results

**Figure 1:**
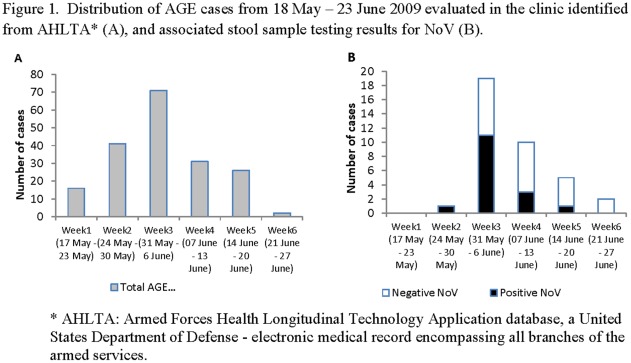
Distribution of AGE cases from 18 May –23 June 2009 evaluated in the clinic identified from AHLTA* (A), and associated stool sample testing results for NoV (B).

From the electronic medical record review from 18 May to 19 Jun 2009, 187 patients presented to the IAB clinic with AGE. The peak incidence of AGE cases was during the week of 31 May –06 June with a total of 71 patients seeking medical care at the clinic (Figure 1), with the outbreak occurring from 26 May to 15 June, 2009. Of the 187, 82 patients completed the case survey, 79% reported diarrhea, 46% reported vomiting, and 29% reported fever (Table 1). The median number of days between symptom onset and clinic visit was 2 days (interquartile range 1, 5). During the 7 days prior to symptoms, 73% of respondents reported travelling off base, 56% reported eating off base, and 24% reported using an outdoor pool.

**Table 1:** Characteristics and activities of gastroenteritis cases reported by case-based surveillance survey responders.

Characteristics	Total; n = 82
Age: Median (IQR)	27(23–35)
Gender: Male, n (%)	51(62)
**Symptoms, n (%)**	
Watery diarrhea	65(79)
Nausea	52(63)
Vomiting	38(46)
Fever	24(29)
Headache	42(51)
Chills	31(38)
Stomach and/or abdominal pain	63(77)
**Base facilities, n (%)**	
Consumption of vegetables, fruits, meat, milk off base	46(56)
Consumption of bottled water	56(68)
Travel off base	60(73)
Self-report of an ill family member	22(27)
Outdoor Pool	20(24)

Stool samples were collected from 37 patients; only 22 of these patients completed the survey questionnaire. Of the 22 stool samples collected along with a completed survey form, 9 were positive for NoV and we could identify their clinical characteristics. Of the NoV positive cases, 44% (4/9) suffered from nausea, 78% (7/9) had watery diarrhea and 67% (6/9) experienced vomiting. No significant difference was noticed between cases positive for NoV as opposed to negative cases in regard to consumption of bottled water, eating and travelling off base, pool usage and presence of a family member with a similar symptom.

Norovirus genogroup II was detected in 43% (16/37) stool samples by RT-PCR. Three of the 16 NoV positive cases were identified with a second enteropathogen: *C. jejuni*, *C. parvum*, or ETEC whereas enteropathogens were recovered from 59% (22/37) of remaining cases (Table 2). With regards to identification of other sole-pathogen infections, 5% (2/37) were positive for *Salmonella* group B, 3% (1/37) were positive for ETEC; 3% (1/37) were positive for *Cryptosporidium*, and 5% (2/37) were positive by EIA for rotavirus; 41% (15/37) of the samples were negative for all pathogens tested (Table 2).

**Table 2:** Distribution of pathogens identified from stool samples (n = 37).

Pathogen detected (Method)	Sole, n (%)	Mixed, n (%)
Cryptosporidium (EIA)	1(3)	1(3)
ETEC (PCR)	2(5)	1(3)
Norovirus (PCR)	13(35)	3(8)
Rotavirus (EIA)	2(6)	0(0)
Campylobacter (culture)	0(0)	1(3)
Salmonella (culture)	2(5)	0(0)

**Figure 2:**
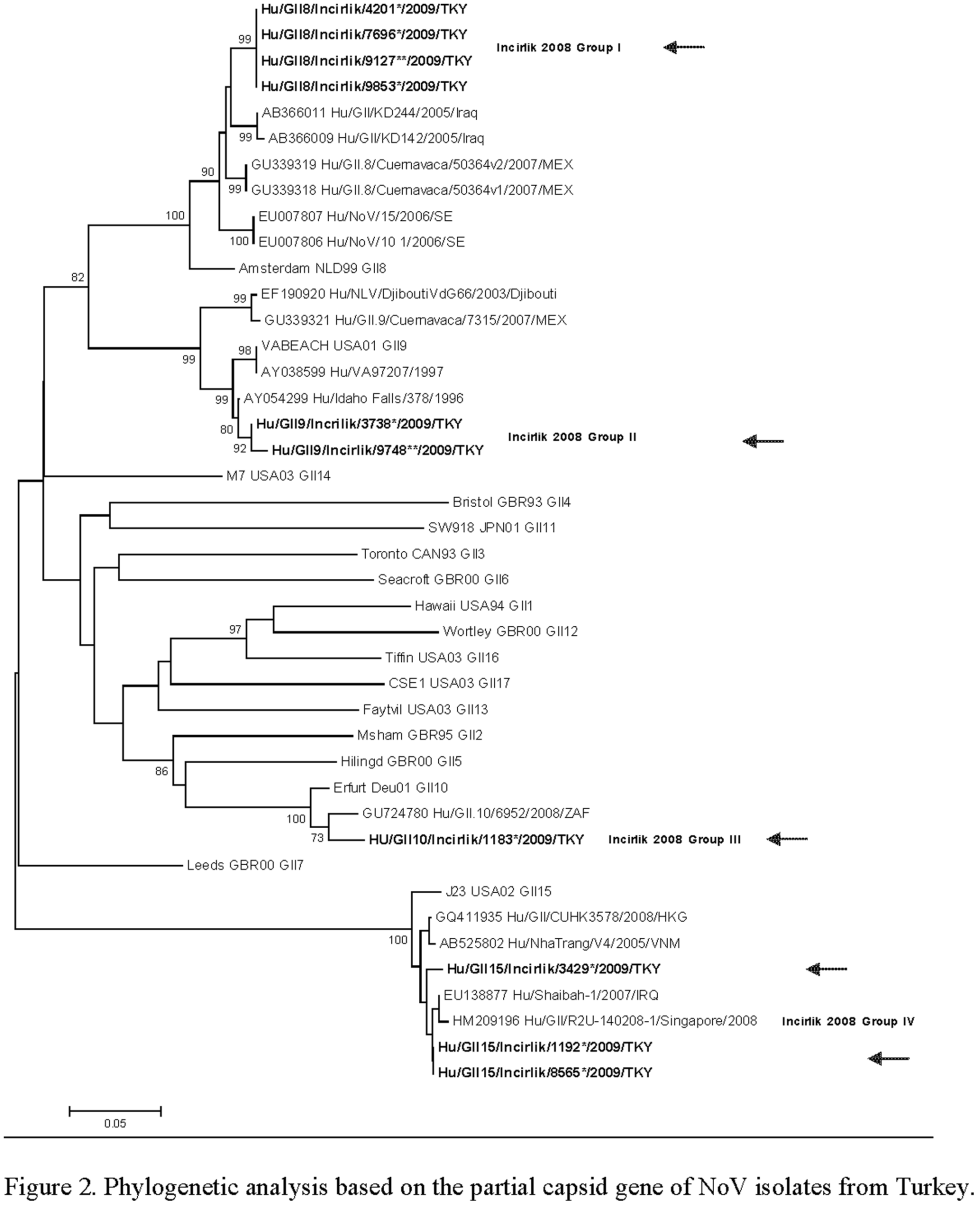
The evolutionary history was inferred using the Neighbor-Joining method. The optimal tree with the sum of branch length  = 2.38 is shown. The percentage of replicate trees in which the associated taxa clustered together in the bootstrap test (2000 replicates) are shown next to the branches. Boot strap values below 70% were considered insignificant and are not shown. The tree is drawn to scale, with branch lengths in the same units as those of the evolutionary distances used to infer the phylogenetic tree. The evolutionary distances were computed using the Kimura 2-parameter method and are in the units of the number of base substitutions per site. The analysis involved 42 nucleotide sequences. Codon positions included were 1st+2nd+3rd+Noncoding. All positions containing gaps and missing data were eliminated. There were a total of 260 positions in the final dataset. Bold sequences represent strains from current study.

We were able to amplify a fragment of the gene encoding NoV capsid protein from 10 of the 16 NoV-positive stool samples; a phylogenetic analysis based on sequence comparison of these products indicated that multiple and rare genotypes of GII NoV were circulating during the outbreak (Figure 2). Four indistinguishable strains (Group I; accession number: JF895615, JF89616, JF973385, JF895613) could be typed as GII.8. A second group of two strains (Group II; accession number: JF895614, JF973386) could be typed as GII.9 and had 99% nucleotide sequence similarity with prototype strain Idaho Falls (accession number AY054299). A third group included one strain that could be typed as GII.10 (Group III; accession number JF895609). Yet another three strains belonged to genotype II.15 (Group IV; accession number: JF895612, JF895610, JF8956111) which had 98–99% nucleotide sequence similarity with other GII.15 strains in the GenBank database.

## Discussion

This report summarizes the results of an AGE outbreak investigation at a US military facility in Turkey that was associated with significant negative operational impact, degrading mission readiness with nearly 20% of the American population in a one month period affected. Initiation of a clinic case-based investigation yielded 37 stool samples in which NoV was detected in 43% (16/37) of the samples with 81% (13/16) of the positive NoV samples identified without a copathogen.

DNA sequencing data demonstrated that several relatively rare genotypes of NoV contributed to this outbreak. Among the 16 NoV positive samples identified, 4 different genotypes were identified. Two of the NoV strains (Shaibah-like, and KD244-like) were previously reported in Iraq and only from deployed troops, while the other two genotypes were reported in South Africa and in the US [25], [26]. In Turkey little systematic data on circulating NoV genotypes exist. However, GIIb/GII.4 strains have been frequently identified in Turkish children with gastroenteritis; strains belonging to this genotype have been found in Europe and mainly in children [27]. Our phylogenetic analysis, although based only on partial sequences from the capsid gene, showed unique strains not previously reported in Turkey. The capsid region of the NoV genome demonstrates great genetic diversity with 20% amino acid differences across capsid sequences [28]. The gene encoding the capsid region is widely used for classification of NoV and provides a reliable marker for genotyping NoV [29]. Homologous recombination, in particular at the junction between ORF1 and ORF2, but also within ORF2 contributes significantly to this high degree of variation [30]. Full capsid sequencing analyses is the gold standard for genotyping of NoV strains, however, for clinical samples with low copy numbers, amplifying and sequence analysis of partial capsid sequences is more practical and is only slightly less discriminatory than full capsid sequencing [31]. In addition, full capsid sequencing has been used to distinguish differences between closely related GII.4 variants [32]; this genotype was not the predominant NoV in our study and partial capsid sequencing sufficiently demonstrated viral relationships.

Previous reports from British troops deployed to Iraq indicated that two NoV strains were responsible for cases of gastroenteritis. Similar mixed NoV outbreaks have been previously observed and are often attributed to systematic failure of cooking/cleaning/drinking water supplies [33]. One limitation of this investigation was that the survey was not used to capture data from a control group (persons without recent AGE); thus we were unable to perform a risk factor analysis. Another limitation was the lack of of environmental samples that could be tested for NoV in order to track the source of outbreak.

To our knowledge a formal outbreak investigation in the Turkish population was never performed. With the reported lack of sensitivity of commercial norovirus EIAs [34], it is important to use molecular diagnostics such as real-time RT-PCR for detection of NoV [35], [36]. Norovirus strain typing of samples collected during outbreaks of AGE in the military is infrequently done. However, with NoV being the most important cause of foodborne illness in the US [2], a full epidemiologic investigation of AGE illness in the military including strain typing of stool samples may be able to limit the magnitude of the outbreak, link unrelated cases to a single contaminated food or water source, and better identify future risks of NoV illness in the military. As a part of the efforts to reduce the risk of outbreaks of NoV gastroenteritis, the US Department of Defense research community is working with US Centers for Disease Control and Prevention to join the electronic NoV outbreak surveillance network CaliciNet [36]. This NoV surveillance network provides centralized reporting of causative NoV strains in the US, epidemiologic information such as likely transmission route, and viral typing information. By combining this existing information new observations, such as seasonality of outbreaks or patterns of emergence of new variants or frequency of food- and waterborne exposure, can be more broadly studied.

NoVs are an important cause of both outbreaks and sporadic disease among military service members. Although usually self-limited, NoV has been associated with significant operational impact. The global significance of the emergence of NoV in recent years as well as its association with military operations, and its divergent molecular epidemiology highlight the need to perform surveillance of circulating NoV infections to characterize and detect evolving strains. This is particularly important now that efforts are underway to develop a NoV vaccine.
